# Draft Genome Sequence of *Desulfovibrio* sp. Strain CSMB_222, Isolated from Coal Seam Formation Water

**DOI:** 10.1128/MRA.00564-21

**Published:** 2021-12-02

**Authors:** Andrew G. McLeish, Paul Greenfield, David J. Midgley, Ian T. Paulsen

**Affiliations:** a Department of Molecular Sciences, Macquarie University, North Ryde, Australia; b Energy, Commonwealth Scientific and Industrial Research Organisation, Lindfield, Australia; c Department of Biological Sciences, Macquarie University, North Ryde, Australia; Loyola University Chicago

## Abstract

Subsurface coal seams contain microbial consortia with various taxa, each with a different role in the degradation of coal organic matter. This study presents the sequenced and annotated genome of *Desulfovibrio* sp. strain CSMB_222, a bacterium isolated from eastern Australian coal seams.

## ANNOUNCEMENT

Coal seams harbor microbial consortia that catabolize organic matter in coal to methane (1, 2). This generation of biogenic methane has important environmental and economic significance. Methane has value as a transition fuel and a source of blue hydrogen (3). Thus, investigation of coal microbes and their roles in coal-to-methane transformations is critical to enhance methane yields and to improve our understanding of subsurface microbiology.

**FIG 1:**
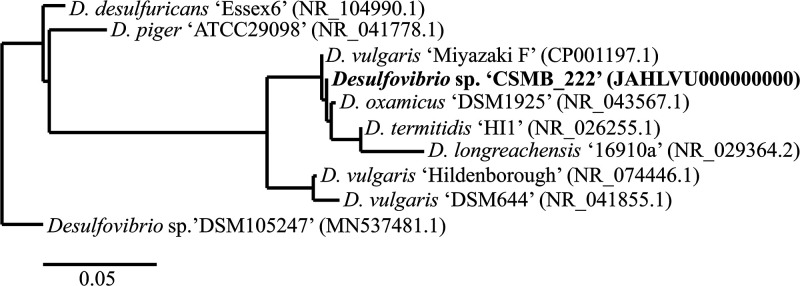
Phylogenetic tree based on 16S rRNA sequences, using a similarity matrix generated by PUP v1.0 analysis (21) (https://github.com/PaulGreenfieldOz/WorkingDogs). The similarity matrix was converted to the Newick format using hierarchical clustering (22) for phylogenetic visualization with TreeDyn v198.3 (23, 24). The numbers in parentheses are GenBank accession numbers.

Formation water from Sydney Basin Well 2 was collected from the Bulli Seam (34°03′S, 150°46′E) at a depth of 650 m (4). This water was anoxically enriched at 40°C, in the dark, using Postgate C medium (5). Dilution to extinction was carried out until an axenic culture of a *Desulfovibrio* species was generated; 16S rRNA sequencing using Earth Microbiome Project primers (6) was used to assess culture purity. Genomic DNA was extracted using a PowerSoil DNA isolation kit (MO BIO Laboratories, Inc., USA), and library preparation was undertaken using the Nextera XT DNA library preparation kit (Illumina) according to the manufacturer’s instructions. The resultant DNA library was sequenced on a HiSeq 2500 system, producing 150-bp paired-end reads (Macrogen, South Korea). The genome sequence data produced 5,291,357 raw reads, which were error corrected using Blue v2.1.4 (7) and assembled using SPAdes v3.13.2 (8), and full-length 16S rRNA was extracted using Kelpie v2.0.1 (9) (Genomic Information *Desulfovibrio* sp. CSMB_222; https://www.doi.org/10.25919/m6yy-fc50). The 16S rRNA sequence matched that of *Desulfovibrio* sp. strain CSMB_222 from the Coal Seam Microbiome (CSMB) data set (4). Phylogenetic analyses of 16S rRNA sequences revealed that *Desulfovibrio* sp. strain CSMB_222 was closely related (16S rRNA identity of >99%) to Desulfovibrio vulgaris Miyazaki F (GenBank accession number CP001197.1), Desulfovibrio termitidis HI1 (GenBank accession number NR_026255.1), and Desulfovibrio oxamicus DSM1925 (GenBank accession number NR_043567.1), and distinct (16S rRNA identity of <96%) from Desulfovibrio vulgaris *sensu stricto,* a clade that includes the type strain Desulfovibrio vulgaris Hildenborough (GenBank accession number NR_074446.1) (Fig. 1).

The draft genome of *Desulfovibrio* sp. strain CSMB_222 was ∼4.2 Mbp, comprising 219 contigs with a mean sequencing depth of ∼50× and a GC content of 66.3%, and was 100% complete, with 0.58% contamination, according to CheckM v1.1.3 (10). The mean, median, and *N*_50_ contig lengths for the assembly were 19,017 bp, 11,315 bp, and 38,319 bp, respectively. The genome was annotated by Prokka v1.14.5 (11), which revealed 3,335 putative protein-coding genes, 55 tRNAs, and 2 rRNAs (https://www.doi.org/10.25919/m6yy-fc50). The genome was examined using TransportDB v2.0 (12) (http://membranetransport.org/transportDB2) and dbCAN v9.0 (13) (http://bcb.unl.edu/dbCAN2) to determine its transporters and carbohydrate-active enzymes, respectively. The taxon possessed limited carbohydrate-active enzymes; in brief, 4 auxiliary activity (AA), 12 glycoside hydrolase (GH), and 4 carbohydrate esterase (CE) enzymes were detected, with no polysaccharide lyase (PL) enzymes. Only 3 carbohydrate-active enzymes with signal peptides were detected (GH13_33, GH23, and GH102). In terms of transporters, the taxon focused on the uptake of amino acids, peptides, and carboxylates, with an extensive array of ABC transporters, tripartite ATP-independent periplasmic transporters (TRAP-Ts), and tripartite tricarboxylate transporters (TTTs). Consistent with the paucity of carbohydrate-active enzymes, the strain possesses a single phosphotransferase system (PTS) and two ABC systems predicted for sugar uptake. Unless otherwise noted, default parameters were used for all software tools.

The *Desulfovibrio* sp. strain CSMB_222 genome contained two CRISPR arrays with 96 spacer sequences, suggesting that viral predation is common, as in many other taxa in coal seam subsurface environments (14–16). The role of this taxon in sulfate-poor coal seams in Australia is unknown; however, it seems likely to be engaged in syntrophy with hydrogenotrophic methanogens, similar to Desulfovibrio desulfuricans (17) and Desulfovibrio vulgaris (18–20).

### Data availability.

This whole-genome shotgun project has been deposited in DDBJ/ENA/GenBank under the accession number JAHLVU000000000, BioProject number PRJNA734375, and BioSample number SAMN19490895. The version described in this paper is version JAHLVU010000000. The raw sequencing reads are available from the Sequence Read Archive (SRA) under BioProject number PRJNA734375.
